# Measurement and Analysis of Human Infant Crawling for Rehabilitation: A Narrative Review

**DOI:** 10.3389/fneur.2021.731374

**Published:** 2021-10-11

**Authors:** Qi L. Xiong, Xiao Y. Wu, Yuan Liu, Cong X. Zhang, Wen S. Hou

**Affiliations:** ^1^Key Laboratory of Nondestructive Testing, Ministry of Education, Nanchang Hangkong University, Nanchang, China; ^2^Department of Bioengineering, Chongqing University, Chongqing, China; ^3^Department of Rehabilitation, Children's Hospital of Chongqing Medical University, Chongqing, China

**Keywords:** infant crawling, measurement, rehabilitation, review, motor development disorders

## Abstract

When a child shows signs of potential motor developmental disorders, early diagnosis of central nervous system (CNS) impairment is beneficial. Known as the first CNS-controlled mobility for most of infants, mobility during crawling usually has been used in clinical assessments to identify motor development disorders. The current clinical scales of motor development during crawling stage are relatively subjective. Objective and quantitative measures of infant crawling afford the possibilities to identify those infants who might benefit from early intervention, as well as the evaluation of intervention progress. Thus, increasing researchers have explored objective measurements of infant crawling in typical and atypical developing infants. However, there is a lack of comprehensive review on infant-crawling measurement and analysis toward bridging the gap between research crawling analysis and potential clinical applications. In this narrative review, we provide a practical overview of the most relevant measurements in human infant crawling, including acquisition techniques, data processing methods, features extraction, and the potential value in objective assessment of motor function in infancy; meanwhile, the possibilities to develop crawling training as early intervention to promote the locomotor function for infants with locomotor delays are also discussed.

## Introduction

Crawling is a four-beat gait, known as most infants' first mobility, and the process of crawling skill acquisition can be interrupted by developmental disorders such as cerebral palsy (CP) (1). Infants with CP usually experience delayed or even lack of crawling skill, which greatly affects the locomotor skill development (2). Based on knowledge of brain plasticity theory (3), with intervention and training at early ages, better prognosis of motor function can be achieved. The positive effects of early intervention on motor development have been verified by a few studies [reviewed by Blauw-Hospers and Hadders-Algra (4)]. In particular, recently developed robotic assistants showed a promising effect in promoting crawling in infancy (5, 6). Furthermore, the motor dysfunctions of infants with CP are due to the neurological injury, affecting its ability to regulate the organization of muscle activity, resulting in abnormal muscle synergistic contractions and kinematic output (7, 8). Thus, objective and quantitative measures of muscle activity and kinematic performance during crawling afford the possibilities to identify those infants who might benefit from early intervention, as well as the evaluation of intervention progress (9, 10), even in infants who cannot walk yet.

**Figure 1:**
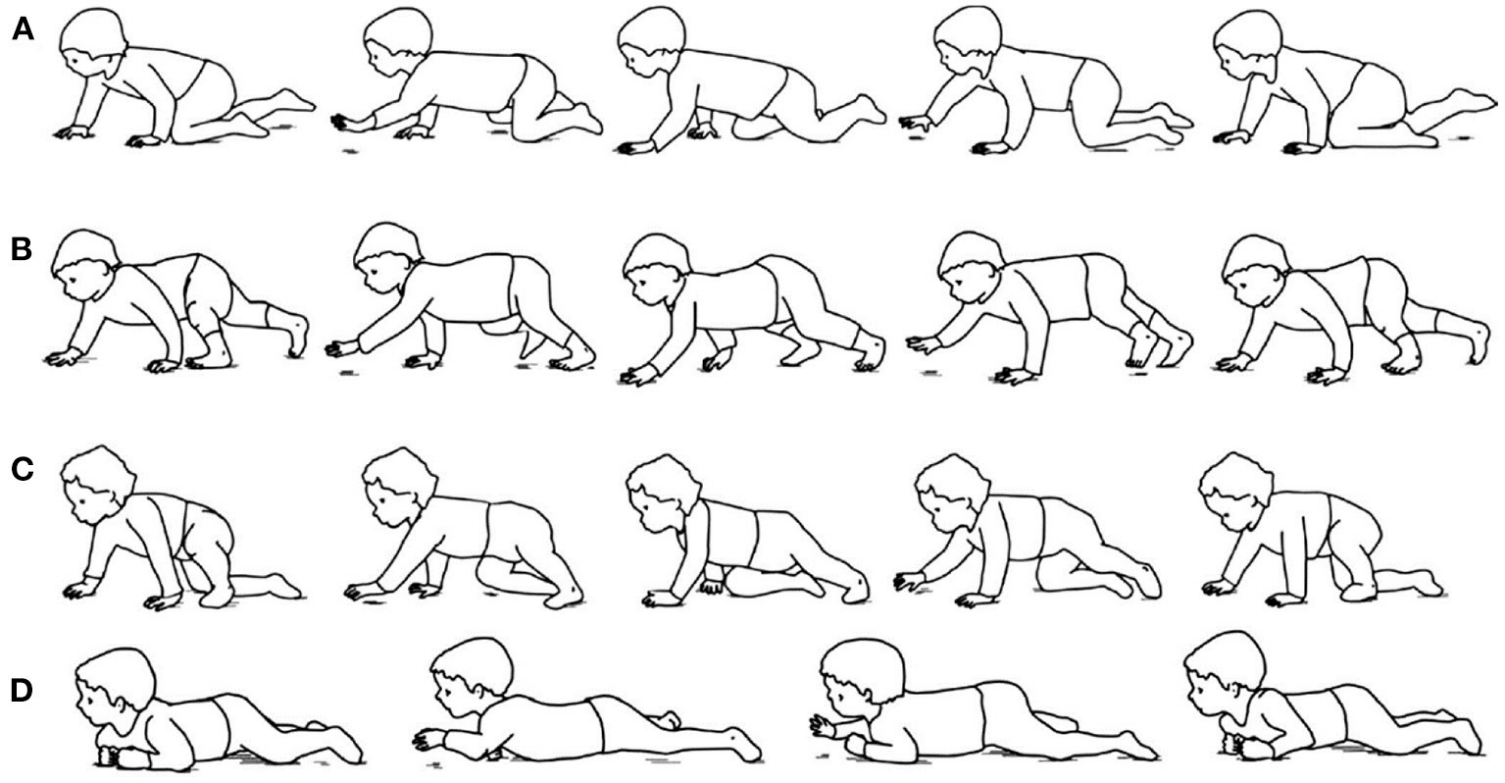
Examples of infant crawling style. **(A)** Hands-and-knees crawling. **(B)** hands-and-feet crawling. **(C)** Step–crawl mix, using foot and right knee. **(D)** Creeping (17).

For those infants without walking ability, traditional assessment tools used by physical therapist provide a measure of delay or abnormality. For example, the amount of delay can be determined by the number of months away from the normal time of milestone achievement (11), but the control mechanisms underlying this delay are not always apparent. Meanwhile, these assessments are relatively subjective and with poor specificity (12). Thus, increasing studies aim to identify objective and quantified measures of infant early movements. For example, quantitative analysis of arm-leg coordination has been performed in neonates' stepping and demonstrated a neural coupling between the arms and legs (13). Then, spontaneous movements around 2 months have been shown to display some primitive forms of coordinative behavior between arms and legs (14). Much later, at approximately the age of 9 months, infant's creeping behavior begins with the clumsy attempts to move forward with the abdomen touching the ground. Subsequently, arms start to develop to have sufficient muscle strength to support the abdomen above the support surface. Then, the typical behavior of hands and knees crawling gradually appears, using the diagonal coordination of the arms and legs (15, 16). In addition, infant crawling includes a variety of atypical crawling postures, such as creeping on the belly, hand–foot crawling, and kneeling-crawling (17), as shown in Figure 1.

It should be noted that infant crawling is quite different from those reported infancy movements, which are either elicited (e.g., stepping) or involuntary (e.g., spontaneous movements). Crawling is a self-motivated rhythmic locomotion that involves the central neural system (CNS)-controlled muscle contraction serving for movement. Thus, quantifying muscle contraction and kinematics during crawling appear to be more likely to uncover the abnormal muscle control strategy that related to the changes/impairments of the CNS (18). With the advancements of wearable sensors and motion capture techniques in recent years, increasing research work has been carried out on the measurement and analysis of infant crawling (19–21). However, to the best of the authors' knowledge, there is still a lack of review work that can provide an overview of the measurement and analysis of infant crawling.

**Figure 2:**
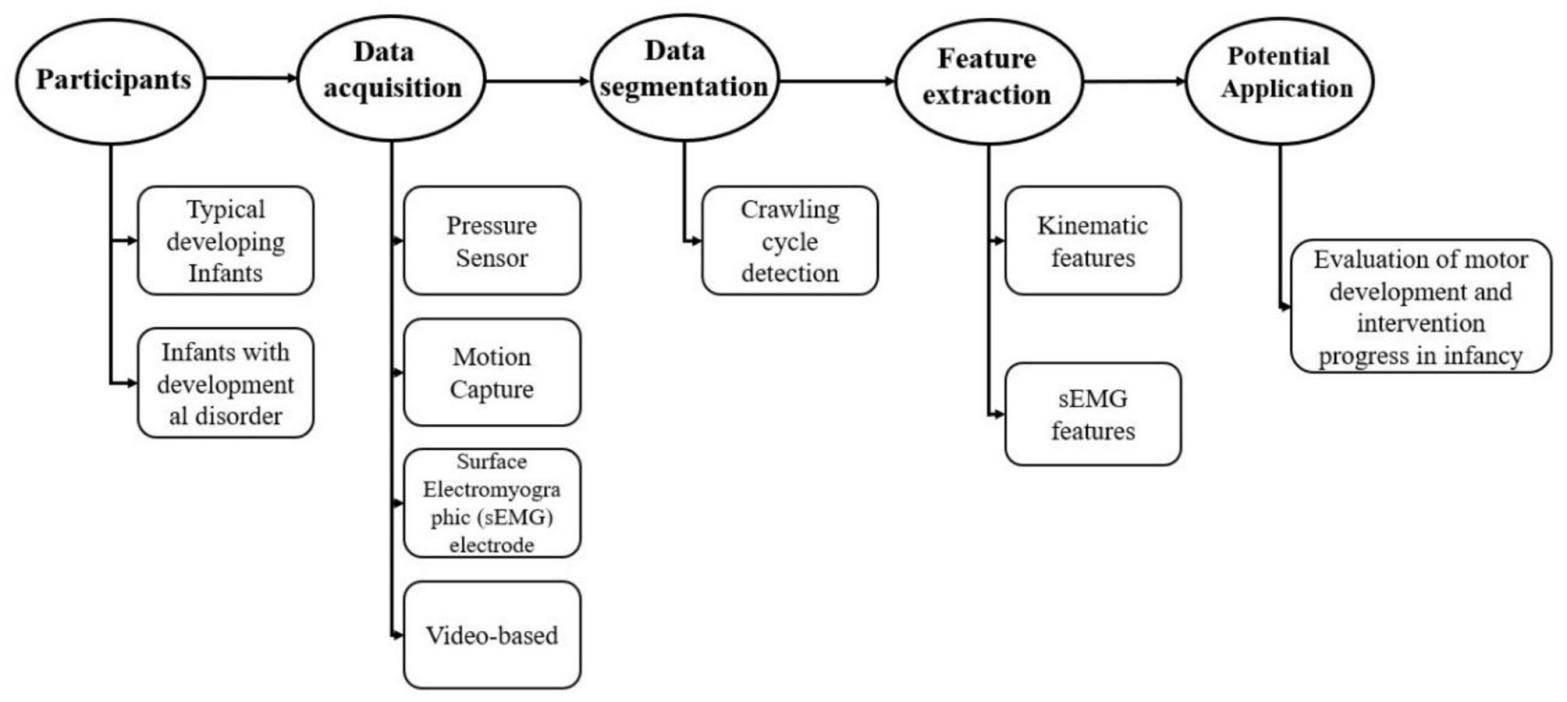
Brief research protocol usually used in the study of infant crawling measures.

To fill this gap, this narrative review aims to give a practical overview of the most relevant measurements and analysis in human infant crawling. In *Data Acquisition of Human Infant Crawling*, we review most of the researches regarding the techniques used for crawling monitoring. In *Detection and Segmentation of Crawling Cycle*, we explore the data processing methodology commonly used to analyze crawling. In *Features of Infant-Crawling Analysis*, we present the main features and indices estimated to characterize crawling movement. In Discussion, the potential values in objective assessment of infant's motor development, as well as the possibility to develop crawling training as early intervention strategy for infants with locomotor delay, are discussed. As shown in Figure 2.

## Data Acquisition of Human Infant Crawling

Early articles are usually based on film records and observational techniques to analyze infant crawling. For instance, early in 1927, two infants were studied using observational techniques to explore the coordination of limbs and body posture during crawling (22). In 1967, 12 crawling sequences were analyzed using graphical methods from six infants, and the differences on the duration of stance between hands and knees were found (23). In 1994, observational and kinematic techniques were used to assess changes of the patterning of the limbs during infant's transition to hands-and-knees crawling (six infants) (16). In 1998, Adolph et al. evaluated infant belly crawling and hands-and-knees crawling by visually analyzing video images at each frame (15). It should be noted that these studies always used visual observation methods and are often anecdotal, which are limited in scope regarding objective measurement of infant crawling, Therefore, Table 1 summarizes recent objective measurement in infant crawling from 2000 to 2021.

**Table 1:** Overview of measurement techniques to monitor human infant crawling.

**References**	**Participants**	**Measurement techniques**	**Placement of marker/electrode**	**Data collected**
Patrick et al. (24)	Healthy infants (*n* = 26)	Motion capture, Electrogoniometer sEMG	Markers: shoulder, elbow, wrist, trunk, hip, ankle Electrogoniometers: hip, knee, shoulder sEMG electrodes: triceps brachii, quadriceps, and hamstrings	Kinematic, joint angle, sEMG
Patrick et al. (17)	Healthy infants (*n* = 22)			
Yozu et al. (25)	Healthy infants (*n* = 8)	Force plate (pressure sensor)	N/A	Vertical peak force
Righetti Ludovic et al. (26)	Healthy infants (*n* = 7)	Motion capture system	Markers: shoulder, elbow, hip, knee, neck, thoracic, lumbar	Kinematic
Ghazi et al. (27)	Not presented	Motion capture	Markers: wrist, ankle, hip, upper back	Kinematic
Xiong et al. (20)	Healthy infants (*n* = 20)	Motion capture system, sEMG	Markers: shoulder, elbow, wrist, hip, knee, ankle, trunk sEMG electrode: triceps brachii, biceps brachii, quadriceps, and hamstrings	Kinematic, sEMG
Xiong et al. (10)	Healthy infants (*n* = 20) Infants with developmental delayed (*n* = 47)			
Gao et al. (21)	Healthy infants (*n* = 17) Infants with CP (*n* = 12)	Motion capture system, sEMG	Markers: shoulder, elbow, wrist, hip, knee, ankle, trunk EMG electrode: triceps brachii, biceps brachii	sEMG oscillations features
Forma et al. (28)	Newborns (*n* = 60)	Motion capture system	Markers: shoulder, pelvis, knees, elbow, wrist, head	Kinematic
Kawashima et al. (19)	Healthy infants (*n* = 16)	Video cameras	N/A	Video-based image

### Pressure Sensors

A pressure sensor usually acts as a transducer; it generates a signal as a function of the pressure imposed. Information derived from such measures is important in gait and posture research for diagnosing lower limb dysfunctions, footwear design, and other applications (29, 30). In the study of infant-crawling measurement, Yozu et al. recruited eight healthy infants to directly crawl on the force plate, which is embedded with multiple single-point pressure sensors. Then the vertical peak ground force (Vpk) was calculated, and no significant difference of Vpk between arm and leg was found when infants were crawling on hands and knees (25). Meanwhile, the pressure data recorded from the pressure sensor placed on children's palm were also used as a reference to detect crawling cycle (31).

### Motion Capture System

**Figure 3:**
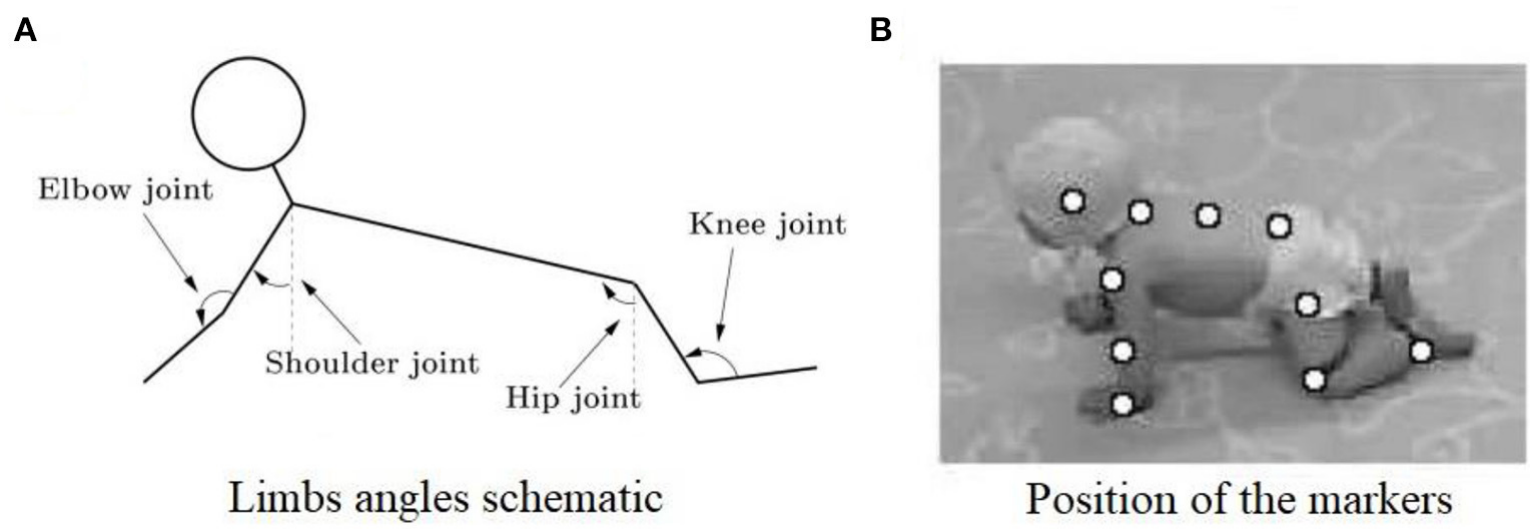
**(A)** Illustration of the measured joint angles in sagittal plane. The shoulder, elbow, hip, and knee joints are measured. **(B)** Snapshot of a crawling infant together with the position of the markers that were used to calculate the different angles (26).

With the rapid development of image processing technology in recent years, motion capture technology has been widely used in human movement analysis. The basic theory of this technique is to record the trajectory of the marker attached on the subjects using multiple cameras, so as to obtain kinematic parameters such as displacement, velocity, and even joint angle (32). In the study of infant crawling, Freedland et al. used video recording and observational techniques to subjectively describe the coordinated movement between limbs (16). In addition, Patrick et al. used a motion capture system to further clarify the prevalence of contralateral coordination of limbs during infant crawling (17, 24), whereas Righetti et al. demonstrated the existence of other limb coordination modes in addition to contralateral coordination pattern in infant crawling and proved that the interlimb coordination pattern in human crawling is consistent with that of other quadruped mammals (26). As shown in Figure 3.

### Surface Electromyography

**Figure 4:**
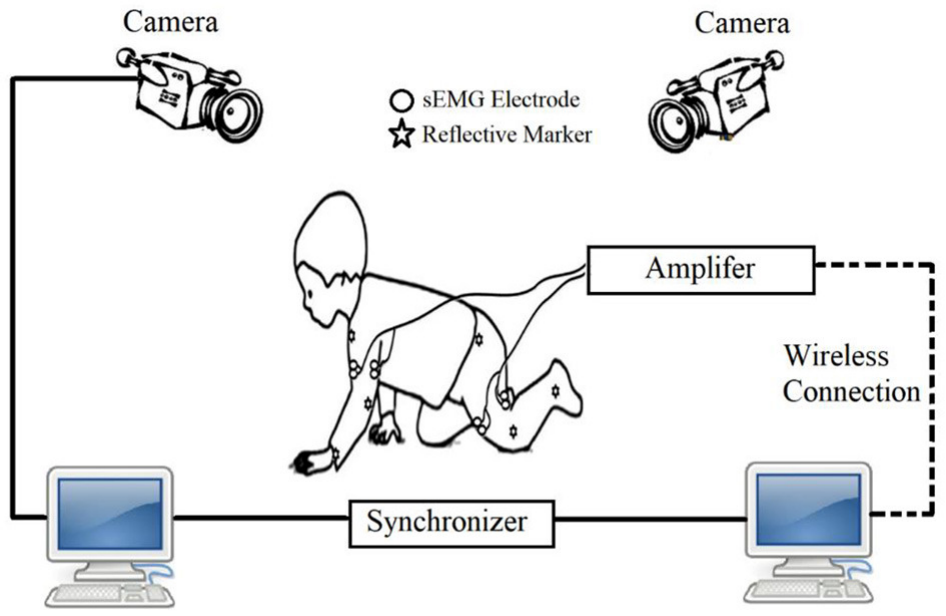
Schematic of crawling measurement based on sEMG and motion capture system.

The surface electromyography (sEMG) is a non-invasive measurement, which mainly uses electrodes attached to the belly of target muscle to collect the electromyographic (EMG) signal that transmitted to the surface (33). Therefore, the sEMG signal can be used to non-invasively monitor the muscle activity during infant crawling. Moreover, sEMG from the triceps brachii, biceps brachii, quadriceps, and hamstring during crawling has been successfully collected in typical developing infants (20) and infants with developmental disorders (10, 21). Specifically, in order to obtain more comprehensive physiological information about infant-crawling movements, recently studies usually combined the aforementioned techniques in the protocol (17), for example, the simultaneous use of motion capture and sEMG acquisition protocol in infant crawling (as shown in Figure 4).

In summary, among the technologies applied to infant-crawling measurement, many researchers have chosen motion capture and sEMG to obtain information of joints and muscle activity. In particular, joint activity measurements are recommended by placing reflective markers at the major joints of the extremities such as shoulder, elbow, knee, hip, and ankle. Meanwhile, the measurements of muscle activity are recommended by placing electrodes on triceps and biceps of the upper extremities, as well as quadriceps and hamstrings of the lower extremities, as these muscles are the major flexion-extension muscles of upper and lower extremities, and the muscle size is big enough to measure in infant subjects. It should be noted that in human locomotion, many muscles are involved, making movement complex and smooth. Therefore, protocol can be improved by recruiting more skeletal muscles such as gluteus maximus. On the other hand, motion capture system and sEMG techniques are required to attach markers or electrodes to the subject's body. For adults and school-aged children, this procedure of data acquisition may be acceptable to the subjects, but for those infants with brain impairment, such as CP infants, the subjects often have emotional abnormalities or lack of voluntary control. The attachment of sensors and markers may cause uncomfortable for the subjects, which may reduce their compliance with the data acquisition process or even refuse to wear the sensors. Moreover, infant crawling is characterized by a high level of natural movement, which is quite different from passive guided crawling in adults (31, 34, 35) or school-aged children (36, 37), who can already walk independently. The attachment of sensors or markers may hinder the most “natural” crawling behavior at both the “physical” and “mental” aspects.

**Figure 5:**
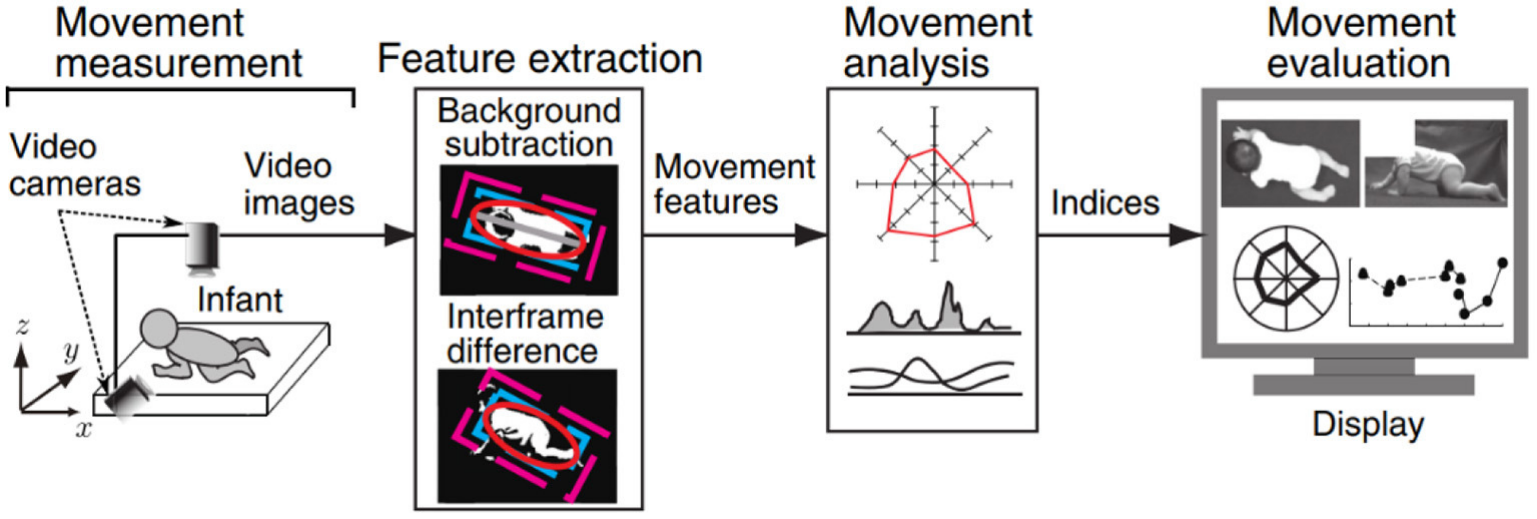
Overview of the computer vision-based measurement system proposed by Kawashima et al. (19).

Computer vision technology uses essentially computers and cameras instead of the human eye to recognize, track, and measure target objects and to obtain limb motion information without placing markers on the target object. This technique has been studied extensively in the field of gait analysis (38, 39), but the application of this technology in the field of crawling motion detection is sparse. Recently, Kawashima et al. verified the possibility of using computer vision–based technology to distinguish different crawling postures from video images for the first time (19). The features of infant crawling were extracted from the recorded video images based on image processing, and then evaluation indices based on video images are calculated for the movement feature extraction and analysis, as shown in Figure 5. The application of this technique can be expected to solve the problem of detecting and analyzing the “natural” crawling mobility of infant subjects.

## Detection and Segmentation of Crawling Cycle

**Figure 6:**
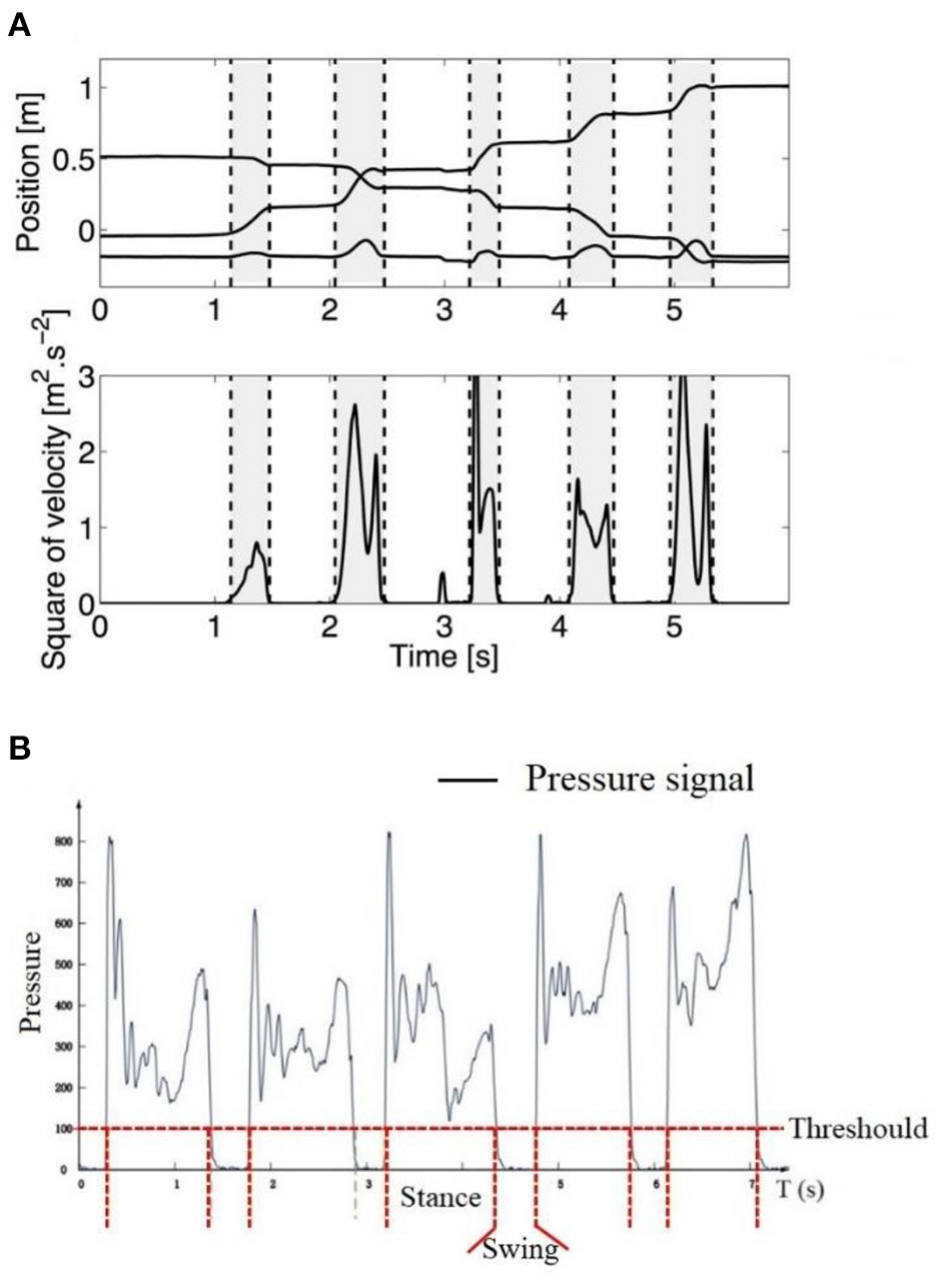
**(A)** Example of stance and swing determined by the trajectory of the wrist (upper graph, the lower line is the vertical direction, whereas the middle and upper one are, respectively, the horizontal and lateral directions). Vertical dashed lines separate swing (gray) and stance (white) phases (26). **(B)** Example of stance and swing determined by pressure threshold. Greater than the pressure threshold for the stance phase and less than the pressure threshold for the swing phase (37).

In order to have comparable data for a steady state of crawling, only the crawling sequences in which the infant was crawling straight toward a goal without stopping were selected, and only complete crawling cycle sequences were considered for feature extraction analysis. A complete crawling cycle was detected from the contact of one limb on the ground to the next contact of the same limb (begins with the onset of swing/stance and ends with the next onset of swing/stance). As the displacement of the wrist in the vertical direction shows rhythmic raising and falling during crawling (as shown in Figure 6A), stance and swing of crawling were usually determined by computing the squared time derivative of the positions (squared of velocity) of the wrist (26). Then, a threshold at 0.5 (*m*^2^/*s*^2^) proposed by Righette et al. was used to decide when the limb was initially moving or stopping (20). In a study on school-aged children's crawling measurement, the stance and swing phase were also determined by the pressure threshold, which was identified by the baseline of the pressure signal collected from the sensor placed on the palm, that is, greater than the pressure threshold for the stance phase and less than the pressure threshold for the swing phase, as shown in Figure 6B.

## Features of Infant-Crawling Analysis

### Kinematic Features

**Figure 7:**
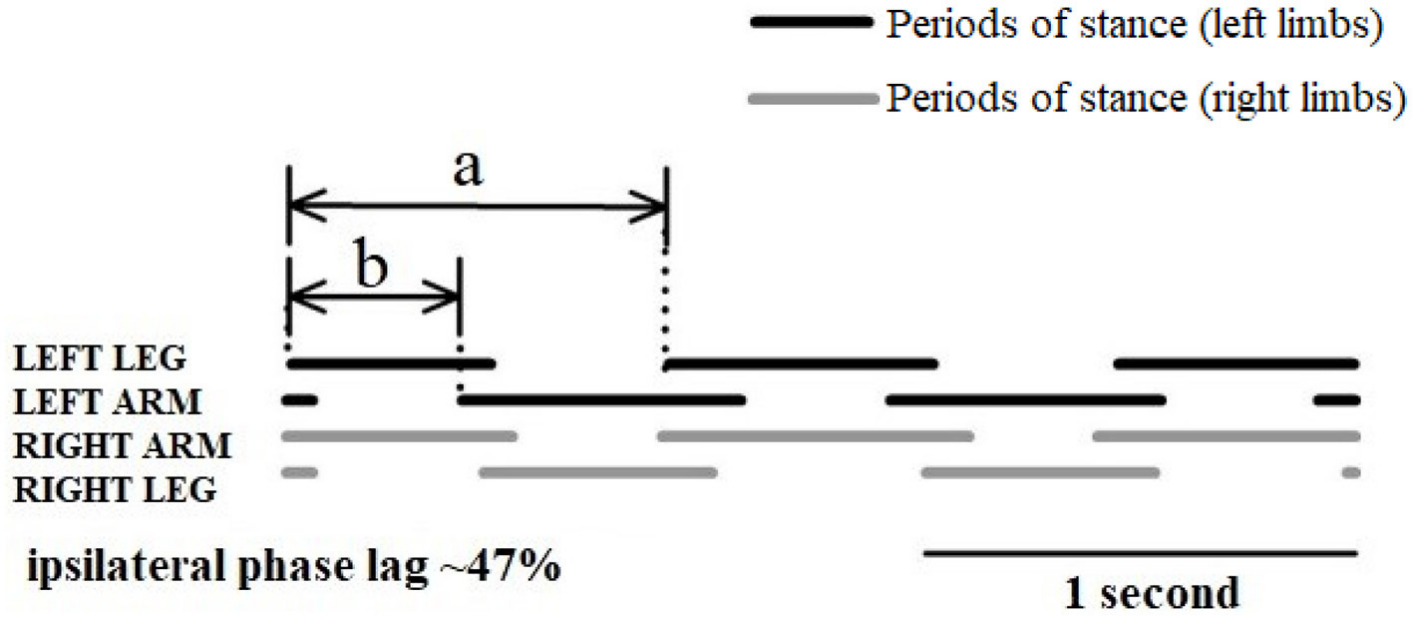
Schematic diagram of interlimb coordination during crawling illustrated with the periods of stance (solid lines) and swing (spaces) (24).

The analysis of infant crawling in the last century focused on the crawling kinematic performance, especially wondering how the limbs coordinate with each other during crawling. To address this issue, Burnside (22) was the first to report that the coordination pattern is between a walking trot and a lateral-sequence walk during crawling on hands and knees. However, there is no clear definition or specific quantification of these interlimb coordination patterns. Then, Hildebrand proposed “the percentage of the step length of the front foot to the step length of the hind ipsilateral” to define interlimb coordination patterns during quadruple locomotion (23, 40). In addition, Patrick et al. studied infant crawling in various conditions (treadmill and normal ground) and proposed the ipsilateral phase lag (IPL) to quantify the coordination of limbs during crawling (24). That is, the relative timing of the right upper limb contact was expressed as a percentage of the crawling cycle determined by consecutive right foot contacts:


(1) $$\begin{array}{l} IPL\;=\;\left(\frac{b}{a}\right)\times 100\% \end{array}$$

where *b* is interval of time between right foot and right hand touchdown events, and *a* is cycle duration (Figure 7). This coordination pattern between the upper and lower limbs may be due to sustained neural connections between the cervical and lumbosacral pattern generators (41, 42). Such connections between the upper and lower limb neural controllers have been verified in human movement by studying the modulation of EMG and reflex activity of upper limbs muscles during rhythmic activity of lower limbs and *vice versa* (42, 43).

In addition to the interlimb coordination during crawling, other kinematic and gait characteristics, such as velocity, duration, and joint angle during crawling, are also of interest. For example, Righetti et al. found a strong correlation between stance duration and crawling velocity when infants were crawling on hands and knees (26). Sparrow et al. further proposed that the duration of the support or swing phase can be quantified by *y* = *a* • *x* • *b* (where *a* and *b* are a constant, *x* denotes the crawling velocity, and *y* denotes the duration of the support or swing phase, respectively) (44, 45). Moreover, Gallagher et al. found that the overall movement durations of the upper and lower limbs during crawling were the same, while the swing phase duration of the upper limb was smaller than that of the lower limb during crawling (46).

### sEMG Features

Crawling movement has been implemented by the CNS-controlled synergistic muscle contraction serving for rhythm limb flexion and extension, and the relevant muscle activities during infant crawling reflect the development status of motor function (18). Possibly due to ethical and technical reasons, quantitative data concerning muscle activities in human infant crawling are limited. In the following section, a few sEMG data collected from children and adults crawling studies were included as well.

#### Muscle Activation Level

The initial interest in muscle activity during crawling is the activation of single muscle, and the parameters such as power, root mean square, and mean absolute value of sEMG signals can well-reflect the activation level of muscle activity. During infant crawling, muscle activation of limbs has been briefly described as triceps brachii is activated throughout the stance phase of the arm during crawling, whereas quadriceps femoris is mainly activated during swing phase of the leg (17, 20). In the study of adult crawling, Maclellan et al. collected the sEMG signals from 26 unilateral upper and lower extremity muscles during adult crawling and found that the activation level of upper extremity muscles was significantly higher than that of lower extremity muscles. Meanwhile, the activation level of lower extremity plantar flexors during crawling was significantly lower compared to upright walking (35). Moreover, the activation levels of the lower quadriceps and tibialis anterior muscles increase with the crawling velocity. Activation level of the triceps and posterior deltoid muscles in the upper limb decreased with the increase of the tilt angle of the crawling plane, whereas the activation level of the gastrocnemius and flounder muscles in the lower limb increased gradually (26). Gallagher et al. compared the muscle activation level of lower extremities when adults were crawling and walking in a constrained space, and they found that the activation level of thigh muscles during crawling was significantly higher than upright walking (46). These findings can be helpful to understand the evolutionary process of the transition from quadrupedal to bipedal walking.

#### Muscle Co-activation

It is well-established that an appropriate level of coactivation between flexor and extensor muscles around joint is required during joint flexion–extension (47). For example, the contraction and stretching of the active muscles make the joints turn in one direction (e.g., flexion), whereas the contraction of the antagonist muscles makes the joints turn in the opposite direction (e.g., extension). The level of muscle coactivation usually been measured by sEMG as the following data preprocessing steps: the raw sEMG data are commonly filtered, demeaned, and rectified, and then the EMG envelope is usually extracted by moving window averaging or low-pass filtering (20). In addition, the coactivation level between antagonistic muscles can be quantified by the coactivation index (CI), which commonly uses the interrelationship between the extracted sEMG envelopes, such as (1) the area of the overlapping region of the envelope curves (48); (2) the area of the product of the envelope curves (20); and (3) the average value of the overlapping region of the envelope curves (49). In the study of infant crawling, Xiong et al. measured sEMG data in 20 healthy infants and demonstrated that characteristics of motor development in infant-crawling stage, for example, non-synchronous development of limbs and rapid reinforcement of the leg, can be manifested by underlying muscle coactivation between flexor and extensor of limbs (20).

#### Muscle Synergy

The generation of coordinated movements of the limbs during human movement requires the participation of multiple muscles, and the aforementioned coactivation of antagonistic muscles can only describe the synergistic activity between two muscles. For the quantification of synergistic contractions across multiple muscles, muscle synergy analysis based on sEMG is a valid tool to explore the coordination across multiple muscles during locomotion (50). It is believed that the CNS might generate motor commands through a liner combination of set of muscle synergies (51), which are the building blocks of synergistic muscle activations coordinated by the CNS to simplify construction of motor behaviors (52).

Specifically, in muscle synergy theory, the data matrix (M) composed of EMG signals from multiple muscle surfaces is often decomposed into a matrix of activation coefficients (C) from the CNS and a matrix of intrinsic muscle synergistic structures (W) present at the spinal cord level (53). Such matrix decomposition is commonly achieved by the non-negative matrix decomposition algorithm, and the most significant feature of this decomposition algorithm is that it can guarantee the non-negativity of the decomposition result, thus assigning the corresponding physiological significance to the decomposed results. The key of the algorithm is to decompose a non-negative matrix M of arbitrary size to obtain two non-negative matrices W and C by iteration, as shown in Equation (2).


(2) $$\begin{array}{l} V^{m\times t}≅W^{m\times r}C^{r\times t} \end{array}$$

where the matrix *V* denotes the original matrix with *m* rows and *t* columns, and *W* denotes the decomposed obtained synergistic matrix with *m* rows and *r* columns in size (usually *r* < *m*); each column in this matrix represents a set of linear combinations (corresponding to the weights of *m* muscles, respectively); *c* denotes the decomposed coefficient matrix, and each row in this matrix represents the activation coefficients corresponding to the above linear combinations. In the past few years, muscle synergy during hands and knees crawling has been studied in healthy infants, young children, and adults. For example, Chen and her colleagues extracted two alternative intralimb muscle synergies from bilaterally limb-related muscles during adult crawling, with one related to the stance phase and the other related to the swing phase. Meanwhile, the structure of synergy was found to be consistent across various crawling speeds (31, 36). In the study of infant crawling, two alternating interlimb muscle synergy patterns were also extracted from eight muscles (bilateral triceps brachii, biceps brachii, quadriceps femoris, and hamstrings) in healthy infants (10). The above findings of muscle synergies extracted from infant and adult crawlers might be suggested as the evidence that there may be two inherent locomotor pattern generators (or neural circuits) that regulate the contractile activity of multiple muscles in infancy locomotor movement from neonates stepping to independent walking (54).

## Discussion

### Quadrupedal Nature of Human Bipedal Locomotion

The same diagonal coupling between the upper and lower limbs during human quadrupedal crawling and walking has been highlighted as evidence that the functional spinal neuronal networks underlying bipedal locomotion have a quadrupedal organization (55, 56). Furthermore, it has been suggested that such arm–leg coordination is due to similar organization of the locomotor pattern generators [usually called central pattern generators (CPGs)] (34, 57–59). Such locomotor networks are operational early in development and exist from neonates to adults. In particular, a recent crawling study on the newborns demonstrated that locomotor CPG network underlying quadrupedal locomotion develops during fetal life (28). Patrick et al. investigated the coordinated pattern of interlimb during crawling in adults and infants, and demonstrated the existence of the CPG network in the intact mature human CNS (24). This abundant evidence of locomotor CPG in humans and the quadrupedal nature of human locomotion raise a question in clinical rehabilitation: To what extent are these locomotor neural networks during quadrupedal locomotion available or reserved after CNS injuries? And, if so, can they be used to promote bipedal walking for those infants with locomotor delayed, such as CP? We briefly discuss these below.

### Crawling Intervention to Promote the Locomotor Function in Early Infancy

The motor dysfunction of CP is one of the most common causes of disability in infants, of which the injury of the developing brain affects multiple networks of the CNS, including corticospinal tract (60) and the physiological state of the spinal cord (61, 62). In particular, if damage to the spinal cord neural network occurs during development, then this can lead to changes in the control of the descending motor pathways and further to change the structure of the spinal cord circuits (63, 64). In addition, most spinal cord synapses are in an inhibitory state (65), and damage to areas such as the cerebral cortex that project to spinal cord interneurons leads to the emergence of an inhibitory–excitatory imbalance in the spinal circuitry, which has been suggested to be a major cause of enhanced segmental reflexes with abnormal radiation of stretch reflexes to other muscles in CP (66, 67).

It is reported that the critical period of maturation of the corticospinal tract is before the age of 2 years (68, 69), Friel et al. even proposed that the neural circuits may be the first to mature in early infancy (3–5 months of age), and once this window is missed, then full recovery of motor function is not possible (62, 64). Thus, early intervention for children with or at risk of CP is critical. It is worth noting that available evidence for early accurate diagnosis of CP can now be made before 6 months' corrected age (70); it is promising to intervene before 6 months in CP. Taking the above considerations for a quadrupedal organization underlying bipedal gait and the idea of critical developmental, we argue that early quadrupedal/crawling training may enhance interventions designed to promote locomotor function for those infants with neurological injuries.

The theoretical justification for quadrupedal training is that using the four-beat gait (i.e., crawling) to elicit walking practice is task-specific for walking, and it incorporates many of the principles of neuroplasticity (71). It addresses the principles of “use it or lose it” and “use it and improve it,” can be implemented with prelocomotor infants, and may affect skill development beyond walking through the principle of transference (72). In developing infant, crawling activates and integrates the different parts of the brain, through crawling, neural connections, and pathways established in the brain. Moreover, the evidence for diagonal coupling in newborn crawling provides additional support for the idea that training crawling at an early age might contribute to the development of upright walking (28). Last but not least, it is worth noting that improvements in gait do not necessarily mean that coordination (or circuitry) necessarily improved, and interventions that increase muscle length or strength can also improve gait without changing underlying coordination (62). For example, the lack of extensor strength due to immature muscle cells is reported as one of the major reasons why human infants cannot walk sooner (62, 73); an important benefit of crawling movement is the enhancement of core musculature, overall strength, and balance in the upper and lower extremities (74). An increase in fine motor skills after a crawling intervention has been found in the subjects with autism spectrum disorder, and younger participants in the study showed the most improvement in their fine motor skills (75). Overall, it is plausible to use crawling intervention as an early rehabilitation technique.

**Figure 8:**
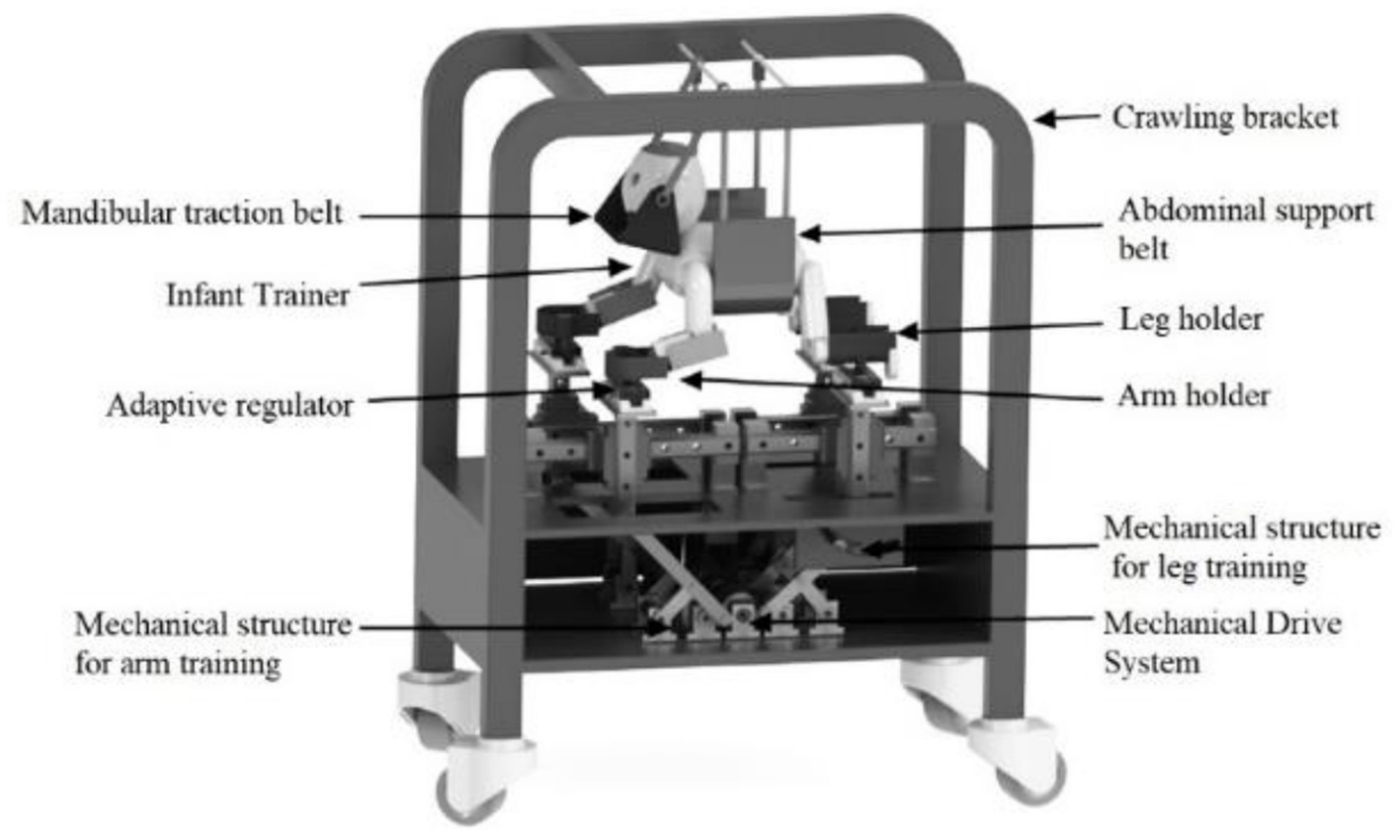
Schematic diagram of crawling training device designed by Jiang et al. (76).

It is relevant to note here that the development of this new rehabilitation strategy is exciting; the supporting scientific evidence for improving gait after crawling intervention remains limited. Nevertheless, several approaches are already reported for implementing early quadrupedal training to hasten the onset of independent walking in children with CP. For instance, a kind of crawling training device was designed to assist children with CP to carry out crawling training (76), as shown in Figure 8. Forma et al. used a skateboard device to elect the newborn crawling behavior (28), and they proved that locomotor circuitry underlying quadrupedal locomotion develops during fetal life, which provides a basis for initiating training in crawling as early as birth. Graessle et al. reported an infant-crawling orthosis to strengthen a neurologically impaired upper extremity (77). Cardenas et al. designed a crawl and gait stimulator that was tested with infants with Down syndrome or CP, and the intervention can significantly shorten the time to complete the path sequence for those infants with motor disorders (78). Some other recent technological assistive solutions for implementing early crawling intervention in children with CP were well-reviewed by Cappellini et al. (62).

### Crawling Measurement as Early Identification of Motor Function in Early Infancy

In addition to early intervention, research is also required to explore motor function changes in response to the intervention, especially given the capacity for the evaluation of intervention progress. For instance, the measurement and quantified metrics of muscle activity during crawling can help to determine whether patients suffer from muscle fatigue and overtraining during quadrupedal training, and kinematic and locomotor metrics can be used to evaluate the effectiveness of crawling intervention on motor function improvement in the long term. As we mentioned in the introduction, objective assessment of motor function during walking can be conducted by gait analysis, which has been widely used in clinics and typically provides quantified metrics of kinematic and muscle activities (79–83). For those infants without walking ability, movement abnormalities are usually assessed by scores obtained on screening tests or visual analysis of their movement quality (84–86), which are relatively subjective and with poor specificity. Thus, measurement of infant crawling after neurological lesions is highly relevant for early interventions in infants at risk of developmental delays (10).

Our previous studies on infant crawling first demonstrated that the sEMG and kinematic parameters such as CI, crawling velocity, and stance phase time during crawling correlated with clinical scale of motor function and thus may be useful in building effective assessment of infant's motor developmental status (20). In addition, the following studies of crawling analysis in infants with developmental disorders supported the hypotheses that muscle synergy indices and kinematic output presented significant differences between infants with confirmed developmental delay and typical developing (TD) infants, whereas the same variables did not show a significant difference between TD infants and infants at risk of development delay (10). Meanwhile, Zhang et al. conducted a preliminary investigation on the difference of interlimb joint synergy metrics during crawling between healthy infants and infants with developmental delay (87), Wu et al. demonstrated the feasibility of using the CIs of biceps, triceps, latissimus dorsi, and triceps during crawling to quantify the relative activation levels in children with CP. Furthermore, they clarified that such CI could be used to evaluate the impairments of bilateral limbs during crawling in children with CP (88). Similarly, Gao et al. proposed that time-varying coefficient curve of sEMG oscillation synergies during infant crawling is a potential index to evaluate the abnormal muscle activities affected by CP disorders (21). Li et al. further explicitly proposed a deviation index integrating sEMG and kinematic features of crawling movements, which was mainly based on the eigenvector ratio to quantitatively assess abnormal crawling functions in children with CP (37). These studies imply a different control strategy during crawling for those infants with different severity of developmental delay, which could be useful to develop more specific, patient-tailored rehabilitation strategy.

In conclusion, two key strengths of the current review are that it (*a*) provides an overview on human infant-crawling measurement and analysis for the first time and (*b*) suggests crawling intervention strategies for improving gait in children with motor developmental disorders. We hope that this article will encourage further thorough investigations performed by the experts from the area of researchers, clinics, and engineering to develop crawling-based intervention strategies/device for improving gait in infants with motor developmental disorders.

## Author Contributions

QX, XW, and WH discussed and contributed to writing the article. QX, YL, and CZ designed and wrote the content of the manuscript. All authors read and approved the final manuscript.

## Funding

This work was supported by the National Natural Science Foundation of China (32000979 and 31971287), National Key R&D Program of China (2020YFC2003800), the Natural Science Foundation of Jiangxi Province (20202BAB216019), and the Science and Technology Project of Education Department of Jiangxi Province (GJJ190512).

## Conflict of Interest

The authors declare that the research was conducted in the absence of any commercial or financial relationships that could be construed as a potential conflict of interest.

## Publisher's Note

All claims expressed in this article are solely those of the authors and do not necessarily represent those of their affiliated organizations, or those of the publisher, the editors and the reviewers. Any product that may be evaluated in this article, or claim that may be made by its manufacturer, is not guaranteed or endorsed by the publisher.
